# Standards of Nutritional Care for Patients with Cystic Fibrosis: A Methodological Primer and AGREE II Analysis of Guidelines

**DOI:** 10.3390/children8121180

**Published:** 2021-12-14

**Authors:** Maria G. Grammatikopoulou, Tonia Vassilakou, Dimitrios G. Goulis, Xenophon Theodoridis, Meletios P. Nigdelis, Arianna Petalidou, Konstantinos Gkiouras, Dimitrios Poulimeneas, Olga Alexatou, Kyriaki Tsiroukidou, Georgios Marakis, Zoe Daniil, Dimitrios P. Bogdanos

**Affiliations:** 1 Department of Nutritional Sciences & Dietetics, Faculty of Health Sciences, Alexander Campus, International Hellenic University, 57400 Thessaloniki, Greece; maria@ihu.gr; 2 Department of Rheumatology and Clinical Immunology, Faculty of Medicine, School of Health Sciences, University of Thessaly, 41110 Larissa, Greece; kostasgkiouras@hotmail.com (K.G.); arianna.petalidou@gmail.com (A.P.); 3 Unit of Reproductive Endocrinology, 1st Department of Obstetrics and Gynecology, Papageorgiou General Hospital, Medical School, Aristotle University of Thessaloniki, 56429 Thessaloniki, Greece; dgg@auth.gr (D.G.G.); meletis.nigdelis@gmail.com (M.P.N.); 4 3rd Department of Pediatrics, Hippokration General Hospital of Thessaloniki, Aristotle University of Thessaloniki, 54124 Thessaloniki, Greece; ktsiroukidou@gmail.com; 5 Department of Public Health Policy, School of Public Health, University of West Attica, Athens University Campus, 11521 Athens, Greece; tvasilakou@uniwa.gr; 6 Medical School, Faculty of Health Sciences, Aristotle University of Thessaloniki, 54124 Thessaloniki, Greece; xenofontheodoridis@gmail.com; 7 Department of Nutrition & Dietetics, Harokopio University, 17676 Athens, Greece; dpoul@hua.gr (D.P.); olga_alexatou@hotmail.com (O.A.); 8 Nutrition and Food Standards Unit, Risk Assessment and Nutrition Directorate, Hellenic Food Authority, 11526 Athens, Greece; gmarakis@efet.gr; 9 Department of Respiratory Medicine, Faculty of Medicine, School of Health Sciences, University of Thessaly, 41110 Larissa, Greece; zdaniil@med.uth.gr

**Keywords:** nutrition intervention, medical nutrition therapy, pulmonary function, critical appraisal, nutrition recommendation, evidence-based dietetics, clinical practice, clinical practice guidelines, pulmonology

## Abstract

Although many Clinical Practice Guidelines (CPGs) have been published for the care of patients with Cystic Fibrosis (CF), including a variety of nutrition recommendations, the quality of these CPGs has never been evaluated. The aim of this study was to compare, review, and critically appraise CPGs for the nutritional management of CF, throughout the lifespan. We searched PubMed, Guidelines International Network (GIN), ECRI Institute, and Guidelines Central for CPGs, with information on the nutritional management of CF. Retrieved CPGs were appraised by three independent reviewers, using the Appraisal of Guidelines, Research and Evaluation II (AGREE II) instrument and checklist. A total of 22 CPGs (seven solely nutrition oriented), by 14 different publishers, were retrieved. The Thoracic Society of Australia and New Zealand CPGs scored the highest overall quality (94.4%), while the Paediatric Gastroenterology Society/Dietitians Association of Australia CPGs had the lowest score (27.8%). Great variation in AGREE II domain-specific scores was observed in all CPGs, suggesting the existence of different strengths and weaknesses. Despite the availability of several CPGs, many appear outdated, lacking rigor, transparency, applicability, and efficiency, while incorporating bias. Considering that CPGs adherence is associated with better outcomes and the need for improving life expectancy in patients with CF, the development of CPGs of better quality is deemed necessary.

## 1. Introduction

Cystic fibrosis (CF) is an autosomal recessive disease that is caused by mutations in the gene for the CF trans-membrane conductance regulator (CFTR), which encodes an ion channel protein, with more than 2000 mutations identified to date [1,2,3]. CF affects appetite, nutritional status, and pulmonary function in a progressive manner, with all synergistically resulting in poor health outcomes [4]. Malabsorption, gastrointestinal dysfunction, genetic modifiers, and chronic and progressive pulmonary infection compromise nutritional status, resulting in growth failure [5,6,7]. As far as nutritional status and pulmonary function are concerned, a two-way relationship exists, with each one affecting the other [8]. As a result, optimizing nutrition is pivotal, with medical nutrition therapy (MNT) being front and foremost in CF management. Recent advances in medicine and supplements [9], multidisciplinary care, the use of more holistic treatment approaches, and adherence to specific dietary protocols have all levelled up the delivery of nutrition intervention among CF patients, reducing growth failure [10,11].

CF-MNT adherence has been associated with ameliorated nutritional status, improved lung function, and better prognosis [12,13]; however, according to the literature, sub-optimal treatment adherence is observed in the majority of patients [14]. On the flipside, CF-specific training opportunities for physicians are limited, especially for adult patients [15], and, as far as clinical practice guidelines (CPGs) are concerned, they exhibit several controversies [16], limiting their adherence and delivery from the physician point of view.

Clear, precise, unbiased, and evidence-based CPGs are needed to promote physician adherence and improve patient prognosis. As such, during the last decade, the quest to compare and appraise CPGs has become a focus. Given that CF-specific CPGs have never been evaluated, the present study aimed to compared, review, and critically appraise CPGs for the nutritional management of CF, throughout the lifespan.

The aim of the present study was (1) to systematically review all CPGs on the nutritional recommendations and CF-MNT for patients with CF, and (2) to critically appraise them. The research question was: What is the quality of CPGs regarding MNT in CF?

## 2. Materials and Methods

### 2.1. PICAR, Search Strategy, Inclusion, and Exclusion Criteria

The PICAR framework, a modification of the PICO(T/S) [17], developed by the University of Ottawa Heart Institute, was applied to shape the research question and define the CPG eligibility criteria [18]. The PICAR strategy applied in the present review is detailed in Table 1.

A systematic search was conducted in PubMed, Guidelines International Network (GIN), ECRI Institute, Guidelines Central, and gray literature, until 2019, aiming to retrieve CPGs and Consensus Statements with information on the nutritional management of CF. The keywords used for the search process included (cystic fibrosis), (nutrition), (clinical practice guidelines), (consensus statements), (nutritional management), (nutritional therapy), (diet therapy), and (pulmonary care).

### 2.2. Inclusion and Exclusion Criteria

Inclusion criteria involved CPGs (1) published in the English language, (2) available in full-text electronic format, (3) for the care of patients with CF, (4) including nutrition recommendations, and (5) intended for healthcare professionals. Exclusion criteria involved CPGs (1) published in languages other than English, (2) for the diagnosis of CF, (3) intended for CF patients, caretakers or family members, (4) lacking nutrition recommendations.

### 2.3. Appraisal of CPGs

Retrieved CPGs were appraised by three independent reviewers using the Appraisal of Guidelines, Research and Evaluation II (AGREE II) instrument [19] and the AGREE II checklist [20]. The AGREE II is a validated tool assessing the transparency and methodological rigor of published CPGs, used in medical and nutrition practice guidelines [21]. Scores were applied in each AGREE domain concerning the scope and purpose of the retrieved CPGs, completeness of stakeholder involvement, scientific rigor, presentation clarity, applicability of the recommendations, and editorial independence. When differences were observed in individual reviewer scores, a fourth reviewer solved the issue after conversation with the review panel. Overall quality scores were calculated for each individual domain and CPG while, additionally, each reviewer advocated for or against the use of specific CPGs for the nutritional management of CF.

### 2.4. CPGs Review and MNT Information

Individual nutrition recommendations were reviewed, categorized, and entered in an excel file by each reviewer independently, to produce an overview of the CF-MNT recommendations.

## 3. Results

### 3.1. Retrieved CPGs and Their Characteristics

A total of 22 CPGs fulfilled the predefined criteria. They were published by the Thoracic Society of Australia and New Zealand (TSANZ) [22]; by a joint committee of the European Society for Clinical Nutrition and Metabolism (ESPEN), the European Society for Pediatric Gastroenterology, Hepatology, and Nutrition (ESPGHAN), and the European Cystic Fibrosis Society (ECFS) [23]; the ECFS alone [24,25,26,27,28,29]; the Cystic Fibrosis Foundation (CFF) [30,31,32,33,34,35,36]; a united effort by the American Diabetes Association (ADA), the CFF and the Pediatric Endocrine Society (PES) [37], a joint committee by the CFF and the North American Society for Pediatric Gastroenterology, Hepatology and Nutrition (NASPGHN) [38]; the Cystic Fibrosis Trust (CFT) [39]; the UK National Institute for Health and Care Excellence (NICE) [40]; the International Society for Pediatric and Adolescent Diabetes (ISPAD) [41]; a joint committee by the Pediatric Gastroenterology Society (PGS) and the Dietitians Association of Australia (DAA) [42]; and the Sociedade Brasileira de Pneumologia e Tisiologia (SBPT) [43]. Table 2 details the main characteristics of the eligible CPGs.

The majority of CPGs were issued by professional organizations and only one was developed by a government authority [40]. They included recommendations for patients of all age groups, including infants, children, and adolescents, as well as adults. Total page numbers ranged from 4 [34] to 768 [40]. Guidelines were published between the year 1995 [34] to 2018 [25].

### 3.2. AGREE Scoring of Included Guidelines

**Figure 1:**
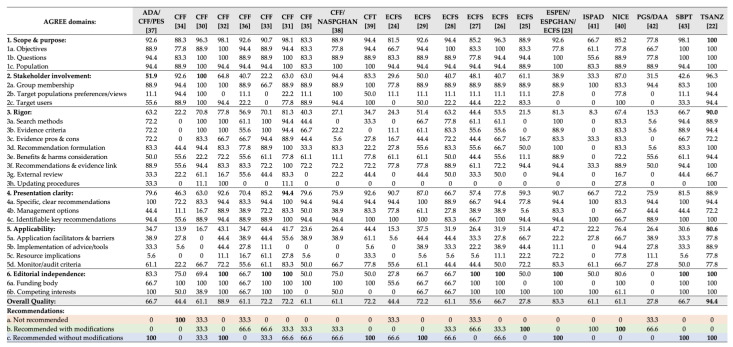
Detailed AGREE II scores of CPGs for the nutritional management of patients with CF (% of maximum scoring for each domain and subcategory). Highest scoring in each category is presented in bold font. ADA, American Diabetes Association; AGREE, Appraisal of Guidelines, Research and Evaluation; CFF, Cystic Fibrosis Foundation; CFT, Cystic Foundation Trust; CPGs, Clinical Practice Guidelines; DAA, Dietitians Association of Australia; ECFS, European Cystic Fibrosis Society; ESPEN, European Society of Clinical nutrition and Metabolism; ESPGHAN, European Society for Pediatric, Gastroenterology Hepatology and Nutrition; ISPAD, International Society for Pediatric and Adolescent Diabetes; NASPGHAN, North America Society for Pediatric, Gastroenterology Hepatology and Nutrition; NICE, National Institute for Health and Care Excellence; PES, Pediatric Endocrine Society; PGS, Paediatric Gastroenterology Society; SBPT, Sociedade Brasileira de Pneumologia e Tisiologia; TSANZ, Thoracic Society of Australia and New Zealand.

Of the 22 CPGs retrieved in total, the TSANZ [22] guidelines obtained the highest score in four out of six main domains of the AGREE II instrument. Subsequently, the TSANZ [22] CPG was suggested by all reviewers for adherence in clinical practice without needing modifications and achieved the highest score in the overall CPGs’ quality assessment. On the other hand, the ECFS [24] CPGs yielded low scores in all domains and the overall assessment, while it was not recommended by one of the experts. Detailed scores received for each domain and subdomain, as well as expert recommendations, are presented in Figure 1.

### 3.3. Nutrition Recommendations in the Included Guidelines

**Figure 2:**
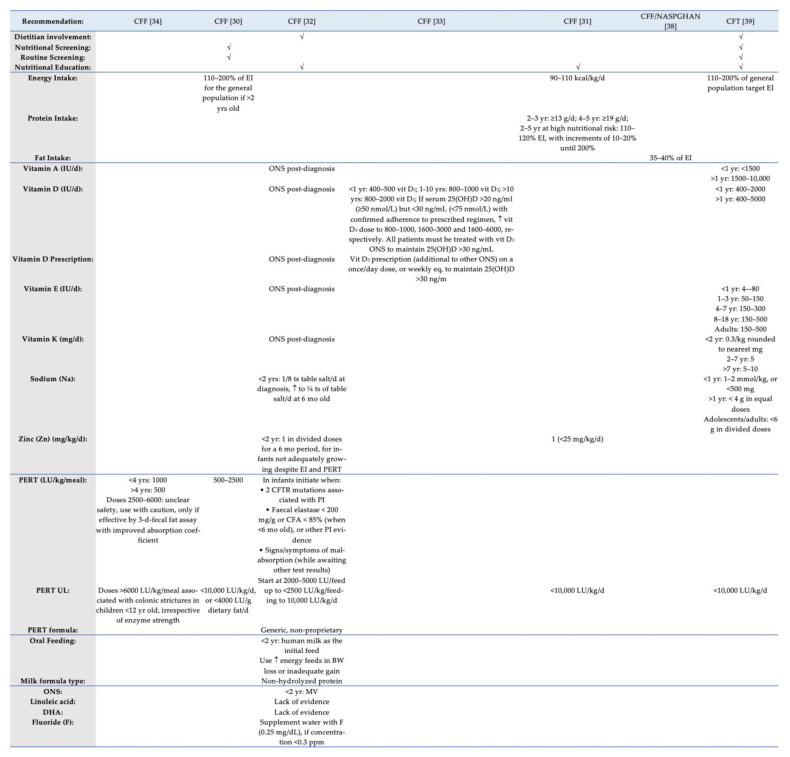
Overview of nutrition recommendations for CF management (part a). BW, Body weight; CF, Cystic Fibrosis; CFA, coefficient of fat absorption; CFF, Cystic Fibrosis Foundation; CFT, Cystic Fibrosis Trust; CFTR, Cystic fibrosis transmembrane conductance regulator; DHA, docosahexaenoic acid; eq, equivalent; EI, Energy Intake; F, Fluorine; LU, Lipase Units; MV, Multivitamin; NASPGHAN, North America Society for Pediatric Gastroenterology Hepatology and Nutrition; ONS, Oral Nutrient Supplements; PI, Pancreatic Insufficiency; PERT, Pancreatic Enzyme Replacement Therapy; ts, teaspoon; UL, Upper Level; 25(OH)D, 25-hydroxy vitamin D; ↑, increase/high.

**Figure 3:**
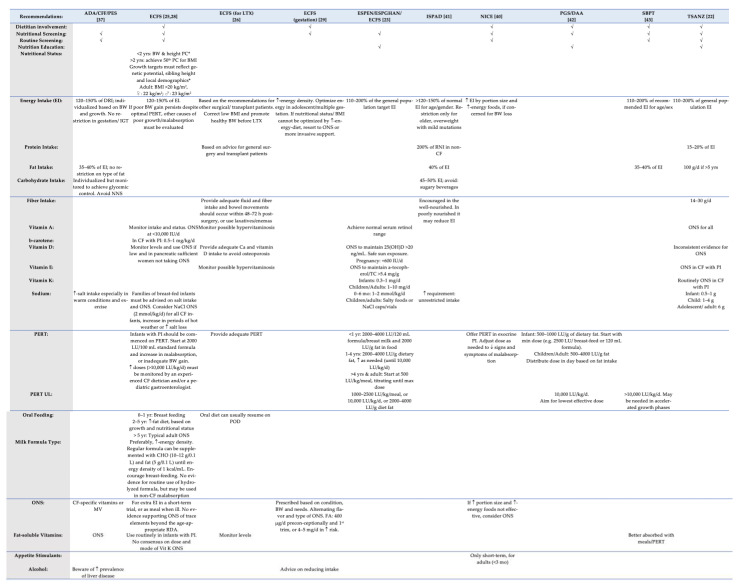
Overview of nutrition recommendations for CF management (part b). ADA, American Diabetes Association; BMI, Body Mass Index; BW, body weight; Ca, Calcium; CHO, Carbohydrate; CF, Cystic Fibrosis; CFF, Cystic Fibrosis Foundation; DAA, Dietitians Association of Australia; DHA, docosahexaenoic acid; DRI, Dietary Reference Intake; ECFS, European Cystic Fibrosis Society; EI, Energy Intake; ESPEN, European Society for Clinical Nutrition and Metabolism; ESPGHAN, European Society for Pediatric Gastroenterology, Hepatology and Nutrition; FA, folic acid; GI, Gastrointestinal; IGT, Impaired glucose tolerance; ISPAD, International Society for Pediatric and Adolescent Diabetes; IU, International Units; LTX, lung transplantation; LU, Lipase units; ΜΝΤ, medical nutrition therapy; MV, multi-vitamin; NaCl, Sodium chloride; NASPGHAN, North America Society for Pediatric Gastroenterology, Hepatology and Nutrition; NNS, non-nutritive sweeteners; NICE, National Institute for Health and Care Excellence; ONS, Oral Nutrient Supplements; PC, Percentiles; PI, Pancreatic Insufficiency; PERT, Pancreatic Enzyme Replacement Therapy; PES, Pediatric Endocrine Society; PGS, Pediatric Gastroenterology Society; POD, post-operative day; RDA, Recommended daily intake; RNI, Recommended nutrient intake; SBPT, Sociedade Brasileira de Pneumologia e Tisiologia; TC, Total cholesterol; TSANZ, Thoracic Society of Australia and New Zealand; UL, Upper level; 25(OH)D, 25-hydroxy vitamin D; ↑, increased/high; ↓, reduced/low; * similar to non-CF.

Figure 2 and Figure 3 present a summary of nutrition recommendations for the management of CF and CF-related complications. Among the 14 advising bodies, the TSANZ [22], CFT [39], and the joint guidelines published by the ESPEN/ESPGHAN/ECFS [23] incorporated the majority of MNT recommendations. On the other hand, the joint committee by PSG/DAA [42] included the least amount of nutrition recommendations.

#### 3.3.1. General Features of the Delivery MNT in Patients with CF

With respect to the involvement of dietitians in the delivery of MNT in patients with CF, several but not all CPGs advocate for the involvement of dietitians. Nutritional screening is an important component mentioned by the majority of CPGs, with the need for routine screening highlighted by some. The need for nutrition education of patients was mentioned by six CPGs only, whereas assessment of patient nutritional status, with sex- and age-specific cutoffs, was provided as a recommendation by the ECFS [25,28].

#### 3.3.2. Energy and Nutrient Intake

As for the adequate provision of energy, most CPGs recommended 110–200% of the respective energy intake of the general population, although different ranges were also suggested (120–150% of the general population goal), mainly for patients with CFRD [25,28,37,41].

With respect to the protein intake, great diversity was presented, with the ISPAD [41] suggesting a consumption equal to 200% of the recommended nutrient intake (RNI), the TSANZ [22] proposing an intake ranging between 15% and 20% of the energy intake (EI), and the CFF [31] suggesting different intakes per distinct age tiers of minor patients.

On the other hand, ideal fat intake appears to be universally more liberal, ranging between 35% and 40% of the EI, irrespective of CFRD diagnosis [37,38,41,43].

As for carbohydrate, recommendations were only provided for patients with CFRD, suggesting an individualized (45–50% of the EI) but monitored consumption, aiming to achieve glycemic control, while avoiding non-nutritive substances and sugary beverages [37,41].

Recommendations for fiber advocated for the encouragement of intake among the well-nourished patients, although, among the poorly nourished, it may reduce energy intake [41]. On the other hand, the TSANZ [22] suggested the intake of 14–30 g on a daily basis. Finally, with respect to patients scheduled for LTX, care should be provided for the adequate intake of fluid and fiber post-surgery, in order to activate bowel movements within 48–72 h [26].

Distinct recommendations were made for different micronutrients, including fat-soluble vitamins (A, D, E, and K), Zinc, and Sodium intake. The intake of Zn is recommended for infants with inadequate growth only [32], whereas adequate Na or salt intake is highlighted by most CPGs, due to increased losses, especially during warm weather conditions and exercise.

With regard to the oral nutrient supplementation (ONS), multi-vitamin (MV) supplements can be prescribed on an individual basis according to the condition, body weight (BW) gain, and personalized needs [29], or when *per os* intake does not appear to promote growth adequately [40]. However, patients should be monitored frequently, especially with regard to the fat-soluble vitamin levels.

#### 3.3.3. Pancreatic Enzyme Replacement Therapy (PERT)

Most of the CPGs provided recommendations regarding PERT, the upper level (UL), formulas, and the ideal dosage in lipase units (LU) and delivery. The UL appears to be estimated at 10,000 LU/kg/day by most CPGs’ panels, although higher doses might be required in accelerated growth phases [43].

#### 3.3.4. Oral Feeding and Formula Type

Breast-feeding should be the initial feed received by the infant [32]. If a milk formula is selected, a high-energy-density formula should be preferred. However, even regular formula can be supplemented with additional carbohydrates (10–12 g/0.1 L) and fats (5 g/0.1 L), until reaching an energy density of 1 kcal/mL of prepared formula [25,28].

#### 3.3.5. Other Issues of Nutritional Concern

According to the NICE [40], appetite stimulants should only be used among adult patients, for a short period of time (<3 months). The intake of alcohol should be reduced, and the high prevalence of liver disease in patients with a CF diagnosis must be highlighted from health care professionals [37].

#### 3.3.6. Provision of EN and PN

**Figure 4:**
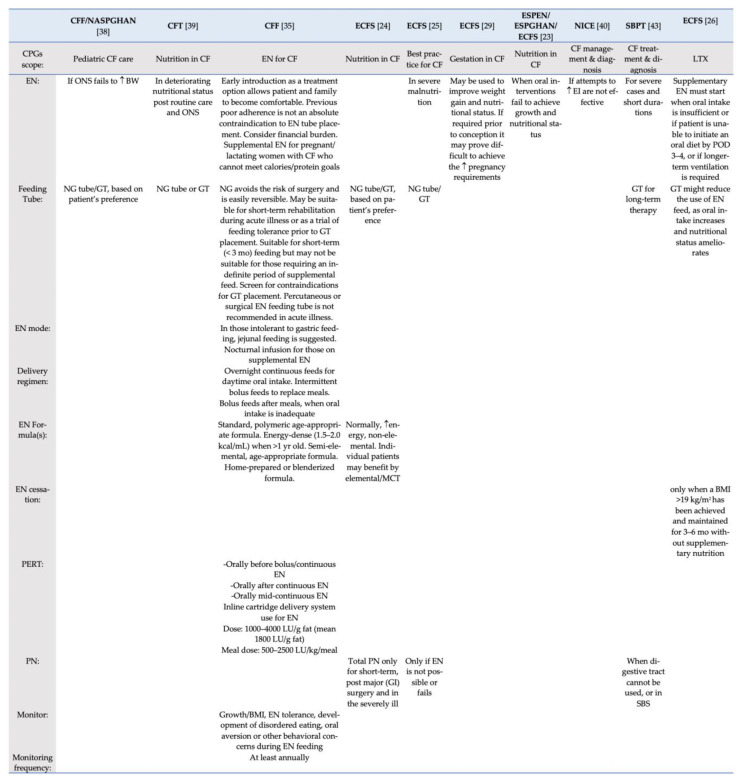
Enteral (EN) and parenteral nutrition (PN) recommendations among included CPGs. BMI, body mass index; BW, body weight; CF, Cystic Fibrosis; CFF, Cystic Fibrosis Foundation; CFT, Cystic Fibrosis Trust; CPGs, Clinical practice guidelines; EI, Energy intake; EN, Enteral nutrition; ESPEN European Society for Clinical Nutrition and Metabolism; ESPGHAN, European Society for Pediatric Gastroenterology, Hepatology and Nutrition; ECFS, European Cystic Fibrosis Society; GI, gastrointestinal; GT, gastrostomy; LTX, Lung transplantation; LU, lipase units; MCT, medium-chain triglycerides; NG, nasogastric; NASPGHAN, North American Society for Pediatric Gastroenterology, Hepatology and Nutrition; NICE, National Institute for Health and Care Excellence; PERT, Pancreatic Enzyme Replacement Therapy; ONS, Oral nutrient supplement; PERT, Pancreatic enzyme replacement therapy; PN, parenteral nutrition; POD, post-operative day; SBS, short-bowel syndrome; SBPT, Sociedade Brasileira de Pneumologia e Tisiologia; ↑, increased/high.

Figure 4 outlines the recommendations regarding the enteral and parenteral nutrition (EN and PN, respectively) included in the CPGs, according to the condition of patients targeted in each CPG. Indications for the use of PN include a short-term provision [24], in severely ill patients or after major gastro-intestinal surgery [24], when the digestive track cannot be used [43] or when EN fails [25].

## 4. Discussion

The present study revealed that many CF-CPGs incorporate MNT information, while seven have CF-MNT as their main aim. Despite the plethora of CPGs, their quality was suboptimal with many methodological limitations identified based on the AGREE, resulting in several CPGs not being suggested for use by the review panel. On the other hand, specific AGREE domains were substantially fulfilled by few CPGs, and few guidelines were recommended without modifications. The highest quality was demonstrated by the TSANZ [22] CPGs, whereas the lowest score was received by the PGS/DAA [42] CPGs, based on the AGREE II scoring system.

The aim of CF-MNT is to maintain growth, well-being, and overall health, limiting symptoms of the disease, while being constantly adapted to either preserve or ameliorate nutritional state [44]. It has been suggested that the aim and purpose of CPGs must be clear and precise, declared early on at the beginning of the document. As such, objectives were clearer in the SBPT [43] and TSANZ [22] CPGs, specific research questions were stressed by the majority of appraised guidelines [22,23,30,31,32,43,45], and target population was defined by many [22,23,30,32,38,39].

On the other hand, stakeholder involvement was adequately reported only by the CFF [30], including the multidisciplinary formation of group membership and the inclusion of target populations’ preferences and views, as well as the inclusion of target users’ (i.e., patients’) involvement. As per patient involvement in particular, with studies indicating a low CPG adherence rate globally [14,46], the need for more patient-centered and patient-involved CF care, becomes evident. Additionally, with dietitians playing an important role in preventing and treating malnutrition, and subsequently disease progression in patients with CF [47], the involvement of dietitians in CF guidelines should not be neglected.

Methodological rigor of development is of great importance in CPGs, as it ensures that recommendations are reliable for decision making [48]. As such, the methodology behind each recommendation suggested should be clearly defined to minimize bias and increase rigor [49]. In the rigor domain, once again the TSANZ [22] CPG received the highest score, accounting for adequate search methods’ definition, grading of evidence criteria, formulation of each recommendation based on the available evidence, and consideration of benefits and harms, while using an external review panel for the evidence analysis and setting a specific update date for the CPGs. The rigor domain revealed several inadequacies in the majority of CF-specific CPGs, with many lacking an external review panel [24,25,42], some underreporting the search methods [24,25,34,38], and few lacking a grading evidence protocol [24,34,39].

Recommendations need to be clear, precise, and unambiguous, as in the case of the CFF [31] and SBPT [43]. Key recommendations, in particular, must be identifiable in the text and management options must be suggested for improved implementation. Furthermore, recommendations must be applicable, with their applicability being facilitated by several tools and audit criteria. It has been suggested that healthcare professionals make better clinical decisions when sound clinical or health policy decisions are facilitated by tools to monitor and implement progress and outcomes. For example, according to healthcare professionals from Spain [50], computer-integrated CPGs might increase physician adherence, whereas in Taiwan [51], positive attitudes were recorded towards computerized CPGs. Overall, more attention is needed on integrating CPGs into everyday practice [52]; however, in the CPGs appraised herein, many were lacking the implementation of tools to facilitate physician adherence [24,30,31,38].

Finally, as far as editorial independence is concerned, the majority of CPGs reported a funding body, with only two lacking relevant information [25,42]. Additionally, competing interests were declared from experts included in the majority of the reviewed CPGs, except from the CFT [39], the ECFS [24], and the PGS/DAA [42]. According to Mozaffarian and Forouhi [53], vested interests tend to influence research priorities and, thus, affect results’ interpretation and relevant recommendations. This is why editorial independence, including conflicts of interest disclosure and declaration of funding, is pivotal in CPGs’ development.

Among all included CPGs, the TSANZ [22] demonstrated the highest overall quality, being followed by the CFF [32] and the ESPEN/ESPGHAN/ECFS [23] ones. Additionally, five CPGs were suggested for implementation by healthcare professionals without modifications [22,23,32,39,43], and one was not recommended at all [34], probably due to outdated methodology and lack of rigor.

Figure 2, Figure 3 and Figure 4 detail individual recommendations for the MNT of patients with CF. According to recent research [44], CF-MNT must be redefined according to age, pancreatic function, and disease stage. As observed by reading the overview tables, the majority of recommendations are age specific, with distinct recommendations being suggested for patients with pancreatic insufficiency. Overall, differences were observed regarding the need for oral nutrient supplementation (ONS), with few nutrients being suggested by some authorities for ONS, while other advising bodies consider the existing evidence as insufficient. Surprisingly, a dietitian is not deemed necessary for CF care in many CPGs, while the need for nutritional assessment and routine screening is also lacking greatly. As Hollander noted [54], with nutrient needs changing dramatically during the disease progress, nutritional care should be personalized and provided by a specialized CF dietitian. Moreover, research has shown that pediatric patients with CF, in particular, are prone to malnutrition [55], often under-consuming several nutrients [56].

As far as energy is concerned, given the reported malnutrition among patients with CF, a more liberal energy consumption is recommended by the majority of CPGs, whereas others suggest enteral nutrition and ONS as a means to manipulate energy intake in cases of inadequate growth. With weight gain being strongly associated with energy and fat intake [57] and many parents relying on energy-dense, nutrient-poor foods to meet the caloric needs of their children [58], more emphasis should be given on the nutrient density of the consumed foods.

Overall, studies indicate that adult patients tend to demonstrate adequate nutrition literacy and confidence in attaining nutrition goals, whereas, as far as children and adolescents are concerned, they exhibit low knowledge scores [59]. In addition, home-based nutrition education programs have shown to be successful in ameliorating nutrition literacy, fat intake, and disease management [60,61]. However, nutrition education does not appear to be of pivotal importance for the majority of advising bodies associated with CF care.

One possible limitation of the present study stems from the exclusion of CPGs published in languages other than the English language. The review and appraisal of CF CPGs, however, is unique, while the focus on nutrition therapy is in accordance with the modern therapeutic approaches for adjuvant CF care.

Today, CPGs’ adherence is considered a quality of care indicator, harmonizing disease outcomes, while minimizing treatment differences between patients of distinct geographic regions and of different socioeconomic status. According to recent research [62], a more uniform care of patients with CF is achieved when implementing clinical pathways for nutrition and lower airway inflammation issues. Based on a nationwide survey, adherence to the guidelines by Australian health professionals has resulted in ameliorated nutritional status among children with CF [63]. In parallel, interventions to increase the degree of adherence to the CF guidelines by patients have resulted in significant improvements regarding nutritional outcomes [64]. Additionally, studies have shown that healthcare professionals are often unaware of the existence of CF-specific CPGs [65]. CPGs’ non-adherence leads to the application of fragmented and inconsistent practices, non-evidence-based clinical decisions, and health discrepancies, impacting the clinical and economic burden of the disease [14]. On the other hand, implementation of CF-related clinical pathways for nutrition and lower airway inflammation issues improves the quality of care, leading to a more uniform management of patients with CF [62].

## 5. Conclusions

In essence, the present study reviewed all existing CPGs on CF care, with a focus on MNT. Despite the existence of several CPGs, many appear outdated, lacking rigor, transparency, applicability, and efficiency, while incorporating systematic bias. Considering that CPGs’ adherence is associated with better outcomes and the need for improving life expectancy in patients with CF, the development of CPGs of better quality is deemed necessary.

## Figures and Tables

**Table 1:** Description of the PICAR strategy.

PICAR Acronym Criteria	PICAR Items Relevant to Screening CPGs for Inclusion
(P) Population	Patients with Cystic fibrosis, throughout the lifespan.
(I) Intervention	Any dietary intervention or MNT for patients with cystic fibrosis (i.e., micronutrient supplementation, provision of energy or macronutrient intake, management of lung transplantation, management of CFRD, etc.).
(C) Comparators, Comparison, and ‘key’ content	Any comparator or comparison. No ‘key’ CPG content is of interest.
(A) Attributes of the CPGs	Eligible CPGs were: (1) CPGs, Practice, or Consensus Papers, (2) published in the English language, (3) in full-text format, (4) until August 2018, (5) from professional or governmental organizations, with (6) nutrition-related recommendations, (7) intended for health professionals, (8) without any limitation in their quality based on the AGREE II.
(R) Recommendation characteristics and other considerations	Not applicable.

AGREE: Appraisal of Guidelines, Research and Evaluation [19]; CFRD: Cystic Fibrosis-Related Diabetes; CPG: Clinical Practice Guideline; MNT: Medical Nutrition Therapy; PICAR: Population, Intervention, Comparator, Attributes, Recommendations [18].

**Table 2:** General description of the included guidelines and their scope.

Advising Body	Year	Origin	Aim			Age Group			Organization		Total Pages
			CF Care with Nutritional Advice	CF MNT	Management of CF Issues, Including Nutritional Care	Infants	Children/Adolescents	Adults	Professional	State	
ADA/CFF/PES [37]	2010	US			CFRD		√	√	√		12
CFF [34]	1995	US	√		PERT	√	√	√	√		4
CFF [30]	2008	US		√			√	√	√		8
CFF [32]	2009	US	√			√			√		21
CFF [36]	2009	US			CFTR-Related MS	√	√	√	√		22
CFF [33]	2012	US			Vitamin D deficiency	√	√	√	√		12
CFF [31]	2016	US	√			√	√				28
CFF [35]	2016	US		√	EN feeding		√	√	√		12
CFF/NASPGHAN [38]	2002	US		√			√		√		14
CFT [39]	2016	UK		√		√	√	√	√		60
ECFS [24]	2002	EU		√		√	√	√	√		25
ECFS [29]	2008	EU	√		Pregnancy			√	√		31
ECFS [28]	2010	EU	√			√			√		7
ECFS [27]	2011	EU			DIOS		√	√	√		5
ECFS [26]	2014	EU	√		LTX		√	√	√		22
ECFS [25]	2018	EU	√			√	√	√	√		26
ESPEN/ESPGHAN/ECFS [23]	2016	EU		√		√	√	√	√		21
ISPAD [41]	2018	International	√		CFRD		√		√		11
NICE [40]	2017	UK	√			√	√	√		√	768
PGS/DAA [42]	1999	AU		√		√	√	√	√		5
SBPT [43]	2017	BR	√			√	√	√	√		27
TSANZ [22]	2017	AU & NZ		√		√	√	√	√		284

ADA, American Diabetes Association; CF, Cystic Fibrosis; CFF, Cystic Fibrosis Foundation; CFRD, Cystic Fibrosis-Related Diabetes; CFT, Cystic Fibrosis Trust; CFTR-Related MS, Cystic Fibrosis transmembrane conductance regulator-related Metabolic Syndrome; DAA, Dietitians Association of Australia; DIOS, distal intestinal obstruction syndrome; EN, Enteral nutrition; ESPEN European Society for Clinical Nutrition and Metabolism; ESPGHAN, European Society for Pediatric Gastroenterology, Hepatology and Nutrition; ECFS, European Cystic Fibrosis Society; ISPAD, International Society for Pediatric and Adolescent Diabetes; LTX, lung transplantation; MNT, Medical Nutrition Therapy; NASPGHAN, North American Society for Pediatric Gastroenterology, Hepatology and Nutrition; NICE, National Institute for Health and Care Excellence; PERT, Pancreatic Enzyme Replacement Therapy; PES, Pediatric Endocrine Society; PGS, Paediatric Gastroenterology Society; SBPT, Sociedade Brasileira de Pneumologia e Tisiologia; TSANZ, Thoracic Society of Australia and New Zealand.

## Data Availability

All data are available upon request to the first author.
